# Effect of Sonication on Microwave Inactivation Kinetics of *Enterococcus faecalis* in Dairy Effluent

**DOI:** 10.3390/molecules27217422

**Published:** 2022-11-01

**Authors:** Ourdia-Nouara Kernou, Amine Belbahi, Yasmine Sahraoui, Kenza Bedjaoui, Kamelia Kerdouche, Akila Amir, Farid Dahmoune, Khodir Madani, Patricia Rijo

**Affiliations:** 1 Laboratoire de Biomathématiques, Biophysique, Biochimie, et Scientométrie (L3BS), Faculté des Sciences de la Nature et de la Vie, Université de Bejaia, Bejaia 06000, Algeria; 2 Department of Microbiology and Biochemistry, Faculty of Sciences, University of M’Sila, M’Sila 24000, Algeria; 3 Department of Biology, University M’Hamed Bougara of Boumerdès, Boumerdès 35000, Algeria; 4 Departement de Biologie, Faculté des Sciences de la Nature et de La Vie et des Sciences de La Terre, Université de Bouira, Bouira 1000, Algeria; 5 Centre de Recherche en Technologie Agroalimentaire, Route de Targua-Ouzemour, Bejaia 06000, Algeria; 6 CBIOS-Centro de Investigação em Biociências e Tecnologias da Saúde, Universidade Lusófona, Campo Grande 376, 1749-028 Lisbon, Portugal; 7 Instituto de Investigação do Medicamento (iMed.ULisboa), Faculdade de Farmácia, Universidade de Lisboa, 1649-003 Lisboa, Portugal

**Keywords:** dairy wastewater effluent, *Enterococcus faecalis*, ultrasound, microwave, inactivation kinetic modeling

## Abstract

The aim of this study is to inactivate *Enterococcus faecalis* ATCC 29212 present in dairy wastewater effluent using microwave (MW) waves and/or ultrasound waves (US). The ultrasonic bath treatment (35 kHz) had no significant effect on the reduction of the survival rate (predominant declumping effect). At 650 W of microwave treatment, the total destruction was completed at 75 s, while at 350 W a 3 log reduction was achieved. The Weibull model was fitted to the survival curves to describe the inactivation kinetics, and the effect of the combined microwave-ultrasound treatments was evaluated. The scaling parameter *α* that was estimated from the inactivation kinetics for the microwaves combined with the ultrasound waves in pre-treatment was found to be lower than the scaling parameters obtained in post-treatment, which were in turn lower than those estimated for microwaves or ultrasound waves alone. The use of the ultrasound waves in pre-treatment was more effective than in post-treatment; a total reduction was achieved using a combination of US (30 min) followed by MW (650 W) with *α* = 28.3 s, while 4.0 log was obtained by reversing all processes with *α* = 34.5 s. The results from the protein assays indicate that the bacterial wall was damaged and that holes were formed from which protein leakage occurred.

## 1. Introduction

The food industry, in particular the dairy sector, is one of the most important sources of wastewater, with a propensity to increase the volume generated. The production of such wastewater is one of the main issues in environmental sustainability. These effluents are subject to microbiological and chemical requirements and can have a significant ecotoxicological impact on aquatic life. In Algeria, a large proportion of wastewater from the food industry (85%) is discharged directly into municipal sewers without any treatment [1].

Considering the high content of organic matter, nutrients such as proteins, and carbohydrates, in addition to the higher concentrations of suspended solids, the Biological Oxygen Demand (BOD) as well as the Chemical Oxygen Demand (COD), wastewater streams, along with substantial pH variability, can contain a multitude of microbiological and chemical pollutants [2,3]. A wide range of microbial profiles have been identified in food effluents, including *Cryptosporidium parvum*, *Giardia sp*., *Escherichia coli*, *Clostridium perfringens*, *Enterococcus faecalis*, *Salmonella sp*., etc. [4,5,6,7,8]. It has been shown that the predominant species in samples from environmental sources (compost, wastewater, sediment, and swimming pool water) are *E. faecalis* (39%) and *E. faecium* (29%), followed by *E. durans*/*E. hirae*, *E. casseliflavus*/*E. gallinarum*, and *E. raffinosus*, with a different prevalence of species depending on the source [9]. These enterococci are an indication of the presence of enteric pathogens [10]. Indeed, disinfection is an important step in wastewater treatment for reducing the pollution levels of the receiving waters and thereby protecting public health.

Treatment technologies commonly used for wastewater include electrochemical treatment, anaerobic treatment, ultrafiltration, chlorination, UV-C irradiation, heat treatment, radiation treatment, and various combinations thereof [11,12,13], such as atmospheric cold plasma [14] and partial denitrification combined with Anammox [15]. Nevertheless, these approaches have drawbacks, i.e., the amount of energy consumed [16], bacterial reactivation [17], the slow metabolic rates of methanogens, a high susceptibility to oxygen concentrations, the complexity of handling biowaste in the case of anaerobic treatment [12], and the influence of environmental factors on the bactericidal effect of cold atmospheric plasma [18]. Several authors have highlighted the use of microwave technology, a green chemistry application with a high sterilization capacity that can effectively inactivate bacteria and enzymes in wastewater [19,20,21,22]. It is a well-known heating and drying process used both for domestic and industrial purposes. Treatment or pretreatment using microwaves provides an increase in the destruction of pathogenic bacteria in the medium, which results in the volume heating of the product and initiates thermal pretreatment [23].

Although the microwave technique has some advantages, some disadvantages remain, and these essentially concern a large consumption of electrical energy being converted into heat. Indeed, given the high specific thermal capacity of water, the decision to use microwaves for wastewater treatment not only implies high energy consumption but also a high cost in operating the system, factors which limit their use. The conventional microwave ovens available on the market provide a high level of disinfection but are not sufficient for use in wastewater sterilization [24].

As a result, another method of disinfection has been the focus of many studies, namely, the use of ultrasound. The effects of ultrasound (US) can be physical (cavitation, mechanical effects, and micromechanical shocks) or chemical, owing to the formation of free radicals (OH- and H- resulting from sonochemical reactions) produced by the decomposition of water within oscillating bubbles [25,26]. Several experiments have explored the use of ultrasound (US) for wastewater disinfection [27,28]. However, in order to achieve a high degree of logarithmic reduction in the microorganisms from the process of ultrasonic irradiation, it is necessary to use a high intensity or a prolonged amount of time. These are the economically limiting factors concerning the application of the large-scale implementation of such disinfection technology. The combining of microwave and ultrasonic methods to reduce the energy needed for successful bacterial destruction is now a practical choice for industry. Leonelli and Mason [29] describe the need to shift towards the optimization of green processes and technologies for the production of microwave and ultrasound reactors on an industrial scale. Therefore, the combination of ultrasound with microwave can induce synergistic effects in terms of efficiency in microbial inactivation, as well as in energy saving [30].

Indeed, the application of this combined technique to the inactivation process for *E. faecalis* (a pathogenic opportunistic bacterium [31] which is considered the most thermoresistant of the vegetative forms [32]) in wastewater is the main purpose of this study, for which we have set the following objectives: (i) to experimentally evaluate the survival of *Enterococcus faecalis* ATCC 29212 cells in wastewater dairy effluent treated by microwave and/or ultrasound, (ii) to fit the Weibull model to describe and compare the inactivation kinetics, (iii) to assess the combined effects of microwave-ultrasound treatment, and (iv) to use extracellular protein assays to study cell membrane integrity.

## 2. Results and Discussion

### 2.1. Ultrasound Effect

**Figure 1:**
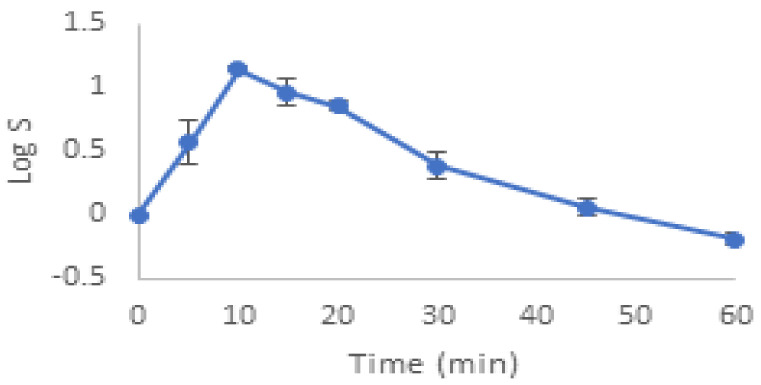
Destruction of *Enterococcus faecalis* ATCC 29121 by ultrasonic bath at a frequency of 35 KHz.

The degradation of *E. faecalis* ATCC 29121 by ultrasonic bath at a frequency of 35 kHz is shown in Figure 1.

The results indicate that there was no significant effect on the viability of the bacterium, with the exception of a small drop in the sonication at 15 min. 

On the other hand, this frequency produced an increase of 1.14 log within the first 10 min, followed by a steady decrease. However, the amount remained higher than the initial concentration even after the 60th minute of sonication, which could also indicate that the declumping effect at this frequency (producing a higher number of CFUs) masked the actual deactivation. The CFU measurement represents cell viability after sonication, although it is important to note that a CFU may be a single cell or a group of cells. Therefore, if ultrasound waves trigger the cells, more CFUs will form.

Obioma [33] revealed that *Enterococcus faecalis* (Gram-positive bacterium with a resistant peptidoglycan cell wall) present in drinking water showed a declumping effect after 2 min of sonication at 20 kHz for a probe. This phenomenon was also observed by Joyce, Phull, Lorimer and Mason [25], who noticed this effect on *Bacillus subtilis* at a frequency of 20 kHz for the probe and 38 kHz in the bath.

Cell death is due to the high pressure and temperature caused by bubble collapse and the shared forces that destroy the bacterial cell membrane [34].

The exposure of high mechanical pressure waves to liquids creates an acoustic current and a subsequent acoustic cavitation that causes the formation, growth, and implosive collapse of micro and nanobubbles in the liquid. These bubbles have a large specific surface area that increases gas diffusion and generates intense localized heating (approximately 5000 °C) and high pressure (1000 ATM) [35,36].

Ultrasonic cavitation affects the inner membrane (cytoplasmic membrane) of the bacteria, and the lipoprotein layer is disrupted, torn, and shredded [37]. *E. faecalis* showed resistance to ultrasonic waves, since it has a thick cell wall. It is well-known that the cell wall of Gram-positive bacteria is thicker than that of Gram-negative bacteria, and this thickness mainly affects the effectiveness of the microbial inactivation [38]. A 4 log reduction in *E.coli* and *E. faecalis* cells present in drinking water was obtained at 9 min, as reported by Gholami, et al. [39], and no declumping effect was observed at 20 kHz. Amabilis-Sosa, Vázquez-López, Rojas, Roé-Sosa and Moeller-Chávez [28] studied the effect of ultrasound on bacterial inactivation in municipal wastewater (MWW); the results showed that, after 15 min of sonication (20 kHz, 35% amplitude and 600 W/l), the bacterial density was reduced by 1.85 Log10 MPN/100 mL for *E. coli* and by 3.16 Log10 CFU/mL for *B. subtilis*. After 30 min, no amount of CFU/mL for *B. subtilis* was observed in the municipal wastewater, and after 45 min, the reduction in total and faecal coliforms was nearly 6.45 Log10 MPN/100 mL.

Various authors have reported on the lethal effects of several bacteria: *Microcystis aeruginosa*, *Circinalis anabaena*, and Chlorella sp. [40]; *Legionella pneumophila* [41], *Pseudomonas aeruginosa*, *Staphylococcus aureus* [42], and *Escherichia coli* [43,44]. In addition, some papers have described the application of ultrasound disinfection technologies [45,46].

### 2.2. Modeling and Kinetic Parameter Estimation

Several mathematical models are suitable for fitting concave downward survival curves [47]; a mathematical model based on the Weibull distribution was chosen for its simplicity and flexibility [48,49].

**Figure 2:**
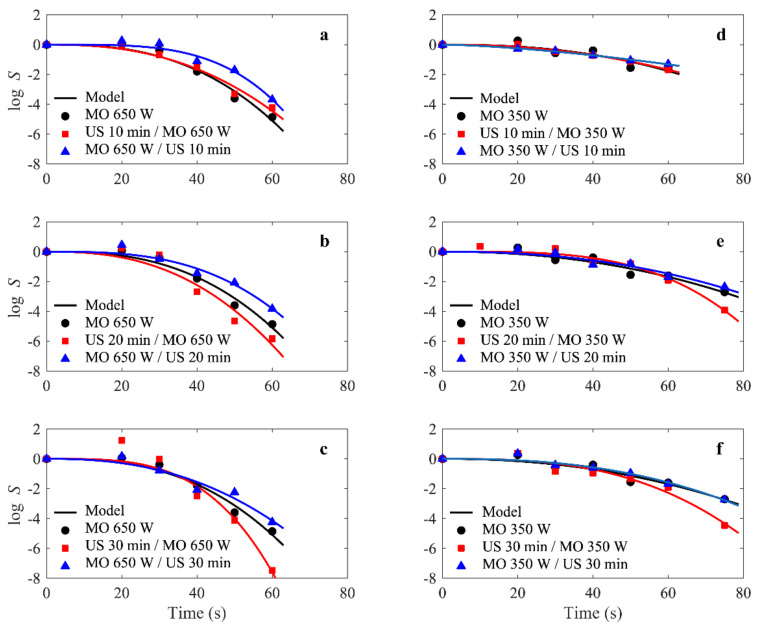
(**a**–**f**) *E.faecalis* survival rate *S*^(t)^ curves under combined microwave (350 and 650 W) and ultrasonic (42 KHz) treatments. Solid lines represent the fit of the modified Weibull model to the experimental *S*^(t)^ data, represented by symbols. The dashed lines are curves added as a visual guide to highlight an increase in survival rate.

In all cases tested in the present study, the modified Weibull model was a very reasonable choice and was well-fitted to the experimental survival data for *E. faecalis* ATCC 29121, as illustrated in Figure 2 (shows the experimental and simulated survival rate *S^(t)^* for *E. faecalis* ATCC 29121 under combined microwave-ultrasonic treatments).

The fit quality was determined by both R^2^ and RMSE values, which ranged from 0.927 to 0.995 and 0.04 to 0.842, respectively (Table 1). This does not mean that other models were not applicable; in fact, other models could also work well. However, the purpose of this paper was not to compare models, but to study the dependence of the Weibull parameters on the power levels and exposure times for microwave and ultrasound treatments.

### 2.3. Microwave Effect

The results show that in the first 10 s at a power of 650 W, there was an increase in the microbial load compared to the initial load, which reached 8.99 ± 0.26 log. This can be explained by the declumping effect; the reduction started from the 10 s mark and reached total destruction after 75 s (Figure 2a–c). On the other hand, a power level of 350 W produced an immediate increase in the CFU with respect to the first second, which reached 8.57 log (followed by a steady decrease). However, the CFU level remained unchanged above the initial concentration, even after 20 s of microwave irradiation, indicating that only the declumping effect can justify this phenomenon. After 75 s, the destruction did not exceed 3 log. Thus, *E. faecalis* ATCC 29,121 was more resistant to lower power than to higher power (Figure 2d–f).

These results suggest that the major effect of a high-powered microwave in the first few seconds is the decommissioning of bacterial agglomerates with little deactivation present. They may also indicate that the downgrading effect at low power levels hides the actual deactivation process. In order to model the results obtained, the inactivation was taken into consideration from the 20th second of microwave treatment. The same results were observed by Benjamin, et al. [50], who studied the effect of microwaves on *Enterococcus faecalis*, *Staphylococcus aureus*, and *Escherichia coli* in water. Their findings revealed that *E. faecalis* appeared to be the most thermally stable of all the bacteria tested (Table 2).

Considering the influence of growth conditions on the shape of survival curves, and in order to quantify the resistance of *E. faecalis* ATCC 29212, the scale parameter (*α*) of each survival curve was re-estimated with a fixed value *β* (*β* = 2.6) for each growth condition. Based on the regression results for the Weibull model, it was found that the *α* parameter generally showed a downward trend and that the *β* parameter tended to increase in value with power levels.

When the survival curves for these two parameters in Figure 2 were compared, it became obvious that the curve with the lowest β value had a more pronounced drag, and the *α* value for the microwave at 350 W was much higher than at 650 W of power.

An increase in microwave power resulted in a decrease in the inactivation rate: at 650 W, the estimated scale parameter *α* (32.2) was the lowest, while at 350 W (50.0), it was the highest, as shown in Table 1.

At MW power levels of 300 W and above (frequency of 2.45 GHz), the disinfection of water was performed when it was heated from 45 to 100 °C. Therefore, the treatment time depended on the MW heating power and the volume of the sample [57]. Ara, et al. [58], reported that the high-level disinfection of enterococci and salmonella is possible in 9.5 min at a temperature of 72 °C reached using microwaves. In addition, microwave irradiation reduced the bacterial content of the sewage sludge prior to anaerobic digestion [59]. Hollywood, et al. [60] reported that a reduction from 3 to 4 log after 7 to 11 min has been obtained from ground beef using a power level of 650 W and suggested that the use of microwave treatment on this strain has a thermal effect and that there was no other factor affecting the inactivation of this strain. *Enterococcus faecalis* was treated at a charge of 10^11^ CFU/mL in a dextrose broth at a power level of 650 W. After 5 min of treatment, survivors were still present, whereas a dosage of 10 min at 650 W was sufficient to destroy all of them. Altogether, the effect of bacterial inactivation from microwave treatment was much more pronounced in a liquid medium than in a solid medium, and as observed by other authors, microbial inactivation was faster when accompanied by an increase in microwave power [61,62].

### 2.4. Combination Effect

It cannot be completely excluded that the inherent inhomogeneity of the dairy wastewater effluent medium, which is caused by milk fat, may pose experimental problems in terms of the regulation of the concentration of this fat in the medium and the agglutination of microorganisms.

The concentration of this fat affects the inactivation of the microorganism, as it has a protective effect on bacterial cells, preventing interactions between bacteria and reactive species (microwave and ultrasound)[14]. Variations in the inactivation of *E. faecalis* ATCC 29212 per microwave and ultrasound treatments were highly dependent on the ultrasound exposure time/power level and the microwave exposure time, as well as on the process chronology (the use of the ultrasound either in pre-treatment or in post-treatment). In all treatments, *β* is greater than one, which determines the value of its concavity parameter downward, meaning that the remaining cells become more and more sensitive to heat. In other words, this indicates that cumulative damage is occurring, making it increasingly difficult for cells to survive.

The values of *α* decreased from 45.1 s for US (10 min)/MW (350 W) to 43.1 s for US (30 min)/MW (350 W), and from 57.3 ± 6 s for MW (350 W)/US (10 min) to 50.5 ± 2.8 s for MW (350 W)/US (30 min). In addition, there is a decrease in the value of *α* at a power level of 650 W, i.e., 33.6, 29.6 ± 2.4, and 28.3 for US 10 min/MW (650 W), US 20 min/MW (650 W), and US 30 min/MW (650 W), respectively. This can be explained by the fact that the more the bacteria are exposed to higher power levels, the stronger the bacterial inactivation effect. These results suggest that the combination of microwaves and ultrasound waves may be useful in improving the inactivation process for *Enterococcus faecalis*.

US pretreatment can increase the efficiency of the microwave inactivation of *E. faecalis* ATCC 29212 in DE. Microwave treatment at 350 W for 75 s combined with ultrasound pre-treatment for 20 and 30 min resulted in a reduction of 3.8 and 4.3 log, respectively, as compared to a reduction of only 2.2 and 3.6 log when sonication was performed as a post-treatment. The same is true for 650 W, where it can be seen that microwave treatment for 60 s followed by ultrasound treatment for 30 min resulted in a 5.0 log destruction, while the total population reduction was achieved when ultrasound was used as pre-treatment.

No study on the subject of this particular technology (the ultrasonic-microwave combination) has been performed; however, studies involving combinations of microwave and ultrasound technology along with other methods of wastewater disinfection have been (Table 2). Blume and Neis [53] evaluated the scientific and economic potential of using an US application as a pre-treatment step in combination with UV to optimize the disinfection process for wastewater; they showed that for 30 s of UV treatment (14 W with 3 W emitted at 254 nm) followed by 5 s of ultrasound treatment at 50 or 310 W/L, the microbial reduction levels obtained were 3.3 log and 3.7 log units, respectively. A synergistic effect was obtained in the elimination of enterococci by US/ozone. Chen, Tang, Wang, Yuan, Wang, Ali and Hu [54], obtained a value of only 1.11 log for enterococcus present in the wastewater after treatment with ultrasound waves at 200 W for 30 min, followed by ozone at a concentration of 4.2 mg O_3_/L; this is still low compared to our results.

However, combined microwave-ultrasound treatment is less important than microwave treatment alone at a power of 650 W; for the following time values: MW (650 W)/US 10 min, MW (650 W)/US 20 min, and MW (650 W)/US 30 min, *α* = 37.8 s, *α* = 36.3 s, and *α* = 34.5 s, respectively. Based on the data obtained from this study, under the same experimental conditions, the ultrasound pre-treatment application had a better effect on the inactivation of *Enterococcus faecalis* ATCC 29,212 than the microwave treatment followed by the US. These results are consistent with the work of Wang, et al. [63].

Since *E. faecalis* ATCC 29,212 has shown a high resistance to microwave treatments and since ultrasound treatments weaken the bacterial wall and contribute to the extraction of intracellular compounds, it could be presumed that the heat generated by microwaves in the liquid and the fragility of the bacterial wall thus accelerate the inactivation and death mechanisms of microbial cells. The results obtained show that a significant reduction in the bacterial population was obtained with a reduced treatment time.

### 2.5. Protein Assays

**Figure 3:**
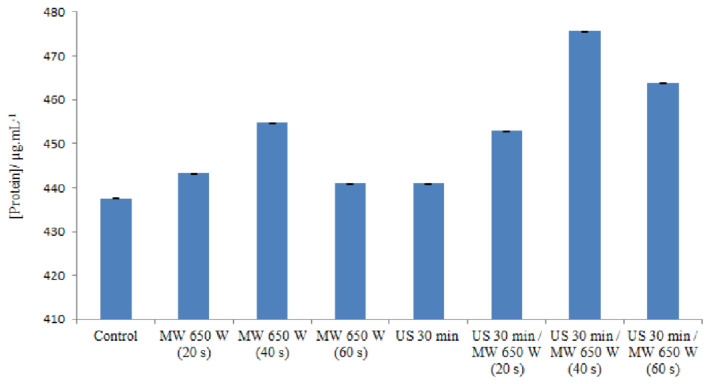
Different concentrations of proteins in different processes in DE.

The release of intracellular proteins was measured in order to study the cell membrane damage caused by microwave and ultrasound irradiation. Figure 3 indicates the various amounts of proteins in different processes.

Microwave heating at 650 W/20 s did not result in a large difference in the amount of protein released from the cells (443.30 μg/mL) compared to the control (437.6 μg/mL). However, when the treatment time was increased to 40 s, substantial differences in the amount of protein released were observed at a value of 454.8 μg/mL. The protein content increased, and these results indicate that most of the microwave-heated cells may be ghost cells with released intracellular material [64]. Indeed, in response to temperature increases as well as chemical or physical stresses, prokaryotic cells synthesize specific proteins involved in cell protection. These stress proteins include a large family called HSPs (which stands for Heat Shock Proteins). Some HSPs are constitutively expressed in cells under normal culture conditions. The induction of a high expression of these proteins occurs when cells are subjected to stress. These proteins are considered “molecular chaperones”: they are thought to be involved in protein folding [65].

A low level of protein leakage was observed when *E. faecalis* ATCC 29212 cells were irradiated for 60 s, a time sufficient for a 3.31 log reduction in the number of viable cells.

On the one hand, some authors propose mechanisms to explain the existence of a stress response under non-thermal conditions. Depending on the power of the microwaves, the effect could be different. Microwave fields could alter the correct folding of some proteins, but not enough to induce a stress response. Misfolded proteins are therefore not protected by the HSP system [66]. On the other hand, according to the principle of the Bradford method, which is used in this study for the determination of proteins, there is a binding (complexation) of Coomassie Blue G-250 with the basic amino acids (arginine, histidine, and lysine) and the hydrophobic residues of amino acids present in the protein(s). The combination of these two explanations leads us to suggest a hypothesis which posits that there would be a decrease in the amount of proteins in the medium when it is at 650 W for 60 s; indeed, after microwave treatment and the refolding of the proteins as explained above, the basic amino acids will not be accessible. Instead, they will be “hidden” in the three-dimensional structure of the proteins with respect to Coomassie blue. This decreases the speed of the basic amino acid–Coomassie blue couple; consequently, the absorbance will be reduced. Nevertheless, according to Woo, et al. [67], who studied the leakage of *Bacillus subtilis* proteins treated using microwaves at 20, 40, and 60 °C, similar results were found and, according to them, the reason for this decrease is still unknown. By using slightly less intense ultrasound waves (35 kHz), it is possible to increase the permeability of the membrane to macromolecules. In this case, the membrane is not broken, only slightly damaged [68], hence an explanation for the low amount of proteins released in the medium. The combined effect from the ultrasound and microwave treatments leads to an increase in the amount of proteins released into the medium; the ultrasound waves weaken the membrane and the microwaves complement the effect of the ultrasound waves by reacting on the membrane.

## 3. Materials and Methods

### 3.1. Preparation of Model Effluent

Model dairy effluent (DE) with a pH value of 6.0 was prepared, as reported by Daverey and Pakshirajan [69], containing 2 g/L semi-skimmed milk powder (Régilait, France), 0.2% (*w*/*v*) milk fat (Ghee, nature foods, Portugal), 0.01% (*w*/*v*) sodium hydroxide (Sigma), and sterile distilled water. The milk powder consisted of 31% protein, 45% carbohydrates, and 14% fat content, with 9% saturated fatty acids and 0.92% salt.

### 3.2. Bacterial Strain and Culture Conditions

Experiments were performed with *Enterococcus faecalis* ATCC 29212 collected from the Pasteur Institute (Algiers, Algeria). The strain was maintained on tryptone soy agar (TSA; Conda Pronadisa, Spain) at 4 °C until use. Young cultures were prepared by suspending colonies in Tryptone Soy Broth (TSB, Conda Pronadisa, Spain) and were incubated at 37 °C for 18 h. Bacterial cells were then recovered by the process of centrifugation (4000× *g* for 15 min at 4 °C) [70,71].

The inoculation of the *E. faecalis* ATCC 29212 strain was adjusted to a final concentration of 1.5 × 10^8^ colony forming units (UFC/mL) in the dairy effluent, as verified by a spectrophotometer (OD_600_ = 0.08–0.13) [72].

### 3.3. Microwave and Ultrasound Treatment Procedure

The ultrasound treatment was performed by the sonication of 40 mL of inoculated DE in an ultrasonic bath (35 kHz) for times ranging between 5 and 60 min. The sample temperature was held at 30 ± 2 °C by gradually adding ice to the ultrasonic bath. Irradiation was conducted in a household microwave (Whirlpool Talent MT263, Malaysia) with a rotating plate (diameter 280 mm) at a speed of 2.46 rpm. The equipment emits a nominal power of 90, 160, 350, 500, 650, 750 and 850 W at 2450 MHz. Inoculated sterile wastewater dairy effluent samples of 40 mL were placed in the microwave cavity and irradiated at 350 and 650 W for different exposure times, ranging between 10 and 75 s. Once treatment was completed, the sample was quickly removed from the cavity and immediately cooled in an ice-water bath. The ultrasound effect as pre-or post-treatment on the microwave inactivation kinetics of *E. faecalis* ATCC 29212 was then investigated.

The combined treatments applied between microwave and ultrasound were: 10, 20 and 30 min for pre- or post-ultrasound treatment and 350 W or 650 W for microwave inactivation kinetics. The experimental procedure was the same as described previously.

### 3.4. Enumeration of Survival Cells

Aliquots (1 mL) of treated and untreated samples were serially diluted in sterile physiological water and spread-plated onto Muller–Hinton medium. The surviving bacterial count was determined after 24 h of incubation at 37 °C. All experiments were performed in triplicate.

### 3.5. Modeling Inactivation Kinetics and Statistical Methods

The survival rate at time *t*, denoted *S*, was calculated as the ratio change for viable *E. faecalis* ATCC 29212 at any time *N^(t)^* (CFU/mL) compared to the initial number of microorganisms *N^(0)^* (CFU/mL) as a control:

(1) $$S=\frac{N^{\left(t\right)}}{N^{\left(0\right)}}$$

The diminution of the log-survival rate data for *E. faecalis* ATCC 29212 obtained in thermo-sonication and microwave treatments was described by the modified Weibull model [48]:

(2) $$\log\left(S\right)=-{\left(\frac{t}{\alpha }\right)}^{\beta }$$

where α is the time of the first decimal reduction and can be called the scale parameter, and *β* is the so-called shape parameter. In order to compare the time of the first decimal reduction for the obtained inactivation kinetics, the Equation (1) was fitted for the second time by setting *β* equal to the mean of the previously estimated value for *β*.

Modified Weibull model parameters were estimated using nonlinear regression with a curve fitting toolbox (MATLAB 6.5, The Math-Works Inc., Natick, MA, USA). The root mean squared error (RMSE) between all the experimental and predicted data, the adjusted coefficients of determination (R^2^), and confidence intervals (calculated with 95% probability) were used as goodness-of-fit indicators for the estimated parameters.

### 3.6. Protein Determination

The protein quantities were estimated according to the method of Bradford [73] with the Coomassie G250 brilliant blue reagent. Both micro and macro assay methods were used.

## 4. Conclusions

In conclusion, the study demonstrated the proof-of-principle for food wastewater safe treatment effluents using ultrasound waves (in pre-treatment) coupled with microwaves for decontamination, with useful efficiency over short treatment periods. The coupling effectiveness varies with the experimental parameters chosen, i.e., the ultrasound exposure time and the power of the microwave. The coupling of ultrasound and microwave treatments was proven to be a promising technological application, as the main indicator micro-organisms in dairy wastewater effluents were reduced and fully inactivated. 

The time values specified for treatment, US 30 min/MW 650 W at 60 s, were sufficient to effectively eliminate heat-resistant bacteria in dairy effluents, while greater resistance to inactivation was presented to microwave only or ultrasound only processing. Therefore, this problem was solved either by extending the duration of the microwave treatment alone, according to the power level, or by combining ultrasound and microwave treatments and, more specifically, by pre-treating with ultrasound, which effectively eliminated the total bacterial load present in the dairy effluents.

## Figures and Tables

**Table 1:** Estimation of the scale parameter (*α*) and shape parameter (*β*) for the fit of survival rate *S^(t)^* for different treatments.

Treatments			*α*	*Β*	*R* ^2^	*RMSE*	*α* (*β* = 2.6)
MW only							
	350 W	-	47.2 ± 9.7	2.2 ± 1.2	0.927	0.314	50.0 ± 4.2
	650 W	-	32.7 ± 7.3	2.7 ± 1.1	0.974	0.375	32.2 ± 1.7
US pre-treatment							
	350 W	US 10 min	47.2 ± 3.4	2.2 ± 0.7	0.981	0.101	45.1 ± 2.2
		US 20 min	50.8 ± 6.0	3.5 ± 1.2	0.975	0.263	45.7 ± 3.6
		US 30 min	45.1 ± 8.5	2.9 ± 1.2	0.953	0.377	43.1 ± 2.8
	650 W	US 10 min	32.8 ± 6.5	2.5 ± 0.9	0.977	0.301	33.6 ± 1.6
		US 20 min	29.1 ± 11.9	2.5 ± 1.6	0.941	0.708	29.6 ± 2.4
		US 30 min	33.6 ± 11.6	3.5 ± 2.3	0.947	0.842	28.3 ± 3.0
US post-treatment							
	350 W	US 10 min	49.9 ± 2.0	1.5 ± 0.2	0.995	0.040	57.3 ± 6.0
		US 20 min	50.6 ± 7.9	2.3 ± 1.1	0.942	0.249	52.4 ± 4.0
		US 30 min	50.0 ± 6.4	2.5 ± 0.9	0.965	0.217	50.5 ± 2.8
	650 W	US 10 min	42.8 ± 6.2	3.9 ± 1.8	0.971	0.291	37.8 ± 3.7
		US 20 min	38.4 ± 7.8	3.0 ± 1.5	0.962	0.344	36.3 ± 2.6
		US 30 min	33.4 ± 9.4	2.4 ± 1.3	0.951	0.413	34.5 ± 2.5

Parameter value ± confidence interval (*p* = 0.05). *RMSE*: Root Mean Squared Error between experimental and estimated data (log *S^(t)^*).

**Table 2:** Ultrasound and microwave irradiation in microbial decontamination technologies for wastewater.

Treatment	Operating Conditions		Micro-Organisms Studied	Microbial Reduction (CFU ml^−1^, UFC/g) Bacterial Survival (%)	References
Ultrasound	350 W.l^−1^/30 min		*Escherichia coli* 0157:h7	1 log UFC mL^−1^	[51]
Ultrasound	20 kHz, 35% amplitude and 600 W/L/15, 30 and 45 min.		*Escherichia coli*	4.55 Log10 MPN/100 mL, 0.48 Log10 MPN/100 mL, 0.48 Log10 MPN/100 mL	[28]
			*Bacilus subtilis*	3.16 Log10 CFU/mL, NA, NA	
			faecal coliforms	5.56 Log10 MPN/100 mL, 3.88 Log10 MPN/100 mL, 0.48 Log10 MPN/100 mL	
			Total coliforms	6.34 Log10 MPN/100 mL, 4.68 Log10 MPN/100 mL, 0.48 Log10 MPN/100 ml	
Ultrasound assisted by UV radiation	1400 W, 15 min	2 UV-C lamps of 150 W	*Escherichia coli*	Below the limits of the legislation	[52]
Ultrasound assisted by UV radiation	30 s of UV radiations (14 W of which 3 W are emitted at 254 nm)	20 kHz, 5 s, 50 W L^−1^ 20 kHz, 5 s, 310 W L^−1^	*Faecal coliforms*	3.30 log CFU mL^−1^	[53]
			*Faecal coliforms*	3.70 log CFU mL^−1^	
US/ozone pretreatment	200 W, 40 kHz at 10, 20 and 30 min	4.2 mg O_3_/L at a flow rate of 600 mL/min	*Enterococci*	0.90 log UFC/g, 0.95 log UFC/g and 1.11 log UFC/g	[54]
			*Total coliforms*	Below 100 CFU/g after 20 min	
Microwave	465 W/30 s, 60 s 1085 W/<30 s		*Escherichia coli*	3 log UFC mL^−1^, <1000 CFU/g <1000 CFU/g	[55]
Microwave	130 W/65 °C		*Enterococcus faecalis*	0.28%	[50]
			*Staphylococcus aureus,*	0.02%	
			*Escherichia coli*	0.04%	
Microwave/anaerobic digestion	60–65 °C/110 s	35 °C, semicontinuous mode for 190 days, V = 4 l, hydraulic retention time was 25 days.	*Faecal coliform*	4.2 log UFC mL^−1^	[56]
			*Salmonella* spp.	Greater than 2 log CFU mL^−1^	

## Data Availability

Not applicable.

## Sample Availability

Samples of the compounds are not available from the authors.
